# A qualitative study exploring the lived experiences of deconditioning in hospital in Ontario, Canada

**DOI:** 10.1186/s12877-021-02111-2

**Published:** 2021-03-09

**Authors:** Sara J. T. Guilcher, Amanda C. Everall, Lauren Cadel, Joyce Li, Kerry Kuluski

**Affiliations:** 1 Leslie Dan Faculty of Pharmacy, University of Toronto, Toronto, Ontario Canada; 2 Institute of Health Policy, Management, and Evaluation, Dalla Lana School of Public Health, University of Toronto, Toronto, Ontario Canada; 3 Institute for Better Health, Trillium Health Partners, Mississauga, Ontario Canada

**Keywords:** Deconditioning, Delayed discharge, Alternate level of care, Delayed transfer, Hip fracture, Hospital, Community, Qualitative, Care transitions

## Abstract

**Background:**

Older adults, especially those with physical and social complexities are at risk of hospital-associated deconditioning. Hospital-associated deconditioning is linked to increased length of stay in hospital, stress, and readmission rates. To date, there is a paucity of research on the experiences and implications of deconditioning in hospital from different perspectives. Therefore, the objectives of this exploratory, descriptive qualitative study were to explore hospital-associated deconditioning from the views of different stakeholders and to develop an understanding of deconditioning from physical, social, and cognitive perspectives.

**Methods:**

Between August 2018 and July 2019, in-depth, semi-structured interviews were conducted with patients 50 years or older, who had a hip fracture or delay in discharge, as well as caregivers, providers, and decision-makers who provided support or impacted care processes for these patients. Participants were recruited from one urban and one rural health region located in Ontario, Canada. All interviews were audio-recorded, transcribed, and analyzed using a constant comparison approach.

**Results:**

A total of 80 individuals participated in this study. Participants described insufficient activities in hospital leading to boredom and mental and physical deconditioning. Patients were frustrated with experiencing deconditioning and their decline in function seemed to impact their sense of self and identity. Deconditioning had substantive impacts on patients’ ability to leave hospital to their next point of care. Providers and decision-makers understood the potential for deconditioning but felt constrained by factors beyond their control. Factors that appeared to impact deconditioning included the hospital’s built environment and social capital resources (e.g., family, roommates, volunteers, staff).

**Conclusions:**

Participants described a substantial lack of physical, cognitive, and social activities, which led to deconditioning. Recommendations to address deconditioning include: (1) measuring physical/psychological function and well-being throughout hospitalization; (2) redesigning hospital environments (e.g., create social spaces); and (3) increasing access to rehabilitation during acute hospital stays, while patients wait for the next point-of-care.

## Background

Patient safety, defined by the World Health Organization as “the absence of preventable harm” [1], is a seminal concept in healthcare and is a focus of policy-makers worldwide [2, 3]. Despite efforts to improve patient safety, recent research suggests that hospital-based patient harm remains an ongoing issue [4–6]. Preventable harm often includes healthcare/medication-associated conditions (e.g., delirium, pressure injuries, medication incidents), healthcare-associated infections (e.g., urinary tract infections, sepsis, pneumonia), patient accidents (e.g., falls), and procedure-associated conditions (e.g., laceration, pneumothorax, device failure) [7]. Preventable harm also includes hospital-associated deconditioning (HAD) [8], also known as post-hospital syndrome or the trauma of hospitalization [9, 10], which is characterized as a period of generalized risk and stress occurring while a patient is receiving care in hospital from an acute condition [9, 10].

HAD has been associated with overall longer lengths of stay in hospital [11], increasing periods of generalized risk and stress [9, 10], and higher rates of readmission [9, 10, 12]. HAD has also been linked to system/organizational factors (e.g., hospital services, systems, policies) [8, 13, 14]. For example, a high stress hospital environment has been shown to contribute to HAD due to sleep disturbances, poor nutrition, limited mobility, and overall uncertainty experienced by patients (e.g., scheduling of care, provider names/roles) [8–10, 12]. To date, the majority of literature on HAD examines risk factors contributing to HAD [8–10, 12, 15], or tools and interventions to assess and limit deconditioning [16–19]. Individual patient factors that increase the risk of HAD include being of older age and having more physical and social complexities [8, 15]. A systematic review of hospital-based interventions targeted at reducing HAD found that while enhanced care programs may be beneficial for certain outcomes (ability to perform activities of daily living), overall, there was low-quality evidence for the risk of physical performance decline, mobility at discharge, readmission rates, length of stay, and mortality at 3 and 12 months when comparing enhanced care programs to usual care [18]. Despite some research on factors impacting HAD and interventions to limit it, there has been little focus on the experiences of deconditioning in hospital from patient, caregiver, and provider perspectives. Given the potential negative impacts of HAD and the limited understanding of individuals’ experiences, our objective was to fill two key gaps in the literature: (1) to explore HAD and transitions in care from multiple stakeholder perspectives and (2) to understand HAD from not only a physical health standpoint, but social and cognitive perspectives as well.

## Methods

### Research design

An exploratory, descriptive qualitative study grounded in naturalistic inquiry was conducted to obtain a description of HAD by exploring experiences of key stakeholders [20]. This analysis was situated in a larger study exploring care transitions in two diverse health regions located in Ontario, Canada. These two settings were selected due to their variation in geography, care delivery, and system indicator metrics [21]. Through the Canada Health Act [22], Ontarians have access to medically necessary, publicly funded hospital and physician care. Despite a core principle of universality, different geographic settings allocate resources differently (e.g., due to variations in bed availability, wait-times for procedures/assessments, and post-acute services) and as a result, experiences may vary within health jurisdictions. The purpose of this analysis was to explore participants’ experiences with activities (physical, social, and cognitive) in hospital and throughout their care transition journey. The Standards for Reporting Qualitative Research were followed [23] and all methods were carried out in accordance with relevant guidelines and regulations.

### Participants

Participants included patients, caregivers (e.g., family, friends), providers (e.g., nurses, rehabilitation professionals, discharge planners), and decision-makers (e.g., clinical managers, leaders within healthcare facilities and regional health authorities). To be included in the study, patients were required to be 50 years or older, English or French speaking, had a hip fracture and/or delay in hospital discharge (known as alternate level of care in Canada) within the past year, and recruited while in one of the two regional hospitals. These two target populations were selected as they are considered more vulnerable, both physically and socially [24, 25]. Caregivers were required to be 18 years or older, English or French speaking, living in the community in one of the health regions, and providing support for someone in the target population. Providers and decision-makers were required to either be providing care or managing care processes for persons in the target populations in one of the two health regions.

### Recruitment and data collection

Participant recruitment started with the main acute hospital, where eligible patients and caregivers were identified by providers. Purposive sampling strategies were used to recruit participants in order to gain a broad range of perspectives from key stakeholders [26]. Hospital staff approached eligible individuals to gauge interest in participation and to obtain consent for the researcher to contact the individual. Patients and caregivers were selected for diversity in target populations (hip fracture and/or delay in discharge, age, gender). Providers were selected for diversity in profession (e.g., nurses, rehabilitation therapists, physicians, discharge planners) and the type of facility/organization in which they worked (e.g., hospital, rehabilitation facility, regional health authority). Snowball sampling methods were also used for recruiting providers and decision-makers by asking participants to identify potential participants at the end of their interview [27].

Three trained researchers (ACE, MSc; JL, BSc; LC, MSc) conducted the in-depth interviews following semi-structured interview guides (one guide per participant type). These researchers received mentorship and ongoing support from the first and senior authors (SJTG, PhD; KK, PhD). For patients and caregivers, the interviews probed on topics relating to experiences in hospital, relationships, formal and informal supports or services prior to hospitalization, health trajectories, discharge plan, and concerns related to discharge. For providers and decision-makers, interviews explored the following topics: role in relation to care transitions, overall experiences in the planning and delivery of care for patients with hip fracture and/or delay in discharge, challenges and success stories, and resources needed to better support discharge processes and care transitions.

Patients were at various points of their care trajectory, with most patients being interviewed in hospital. Interviews were conducted both in-person and by telephone, depending on participant preferences. Written or verbal consent was obtained from all participants (both approaches for consent were approved by the Research Ethics Boards). All interviews were audio-recorded, transcribed verbatim, and cleaned of identifiable information for analysis. Each participant was assigned a pseudonym, which are used in the results section to present the quotes. The interviewer completed reflexive notes immediately following each interview to reflect on key concepts from the interview and areas to probe further in the future.

### Data analysis

Analysis of interview transcripts was conducted concurrently with data collection until data saturation was achieved [28]. Components of the Qualitative Analysis Guide of Leuven [29] and the framework method [30] were applied to ensure a rigorous process. Transcripts from different types of participants were reviewed independently for key concepts and were discussed during weekly meetings to gain a holistic understanding of participants’ experiences. The key concepts informed the development of a codebook, which three researchers (ACE, JL, LC) independently applied to four interviews (one from each type of participant, split between the health regions) using NVivo 11. Coding was compared during in-person meetings (98% agreement), differences were discussed and resolved through consensus, and minor revisions to the codebook were made. Changes to the codebook were discussed and approved by the research team. All transcripts were manually coded in NVivo 11 using the updated codebook (ACE, JL, LC). Data trustworthiness and validity checks were conducted to provide assurance of data quality and rigor (e.g., conducting coding comparisons, re-occurring check-ins during the coding, and analysis processes) [31]. Using the coded data related to activities, display matrices were applied to compare data both within and across regions [32]. Data were stratified by multiple variables (e.g., participant type) to compare and contrast findings and to identify core categories, patterns, and relationships in the data.

## Results

In total, 80 individuals participated in 109 interviews between August 2018 and July 2019. The number of participants (urban: 42; rural: 38) and interviews (urban: 56; rural 53) were evenly distributed across the two regions (see Table 1). Most patient participants had multi-morbidities (e.g., dementia, heart disease, diabetes, cancer, kidney failure). Many patients also experienced social complexities including precarious housing, financial instability, and limited caregiver support. Caregivers were almost exclusively family members, most frequently a spouse or child.

**Table 1:** Participant Demographics (*n* = 80 unique participants)

	Participants			
	Hip Fracture	Delay in discharge	Both	Total
**Urban Region**				
Patients	7	7	1	15
Caregivers	4	9	2	15
Providers^a^	–	–	6	6
Decision-Makers^a^	–	–	6	6
**Rural Region**				
Patients	5	8	2	15
Caregivers	–	3	4	7
Providers^a^	–	–	11	11
Decision-Makers^a^	–	–	5	5

^a^ Providers and decision-makers worked with both hip fracture and delay in discharge patients

We compared experiences between the regions and found that the experiences and challenges around HAD were largely similar. Three main categories were identified, which were applicable to both urban and rural settings: (1) low level of activity in acute care (social, cognitive, physical), (2) tensions around patient identity changes and care transitions’ uncertainty, and (3) physical and social context of deconditioning (social capital, hospital’s built environment). Several sub-categories were identified within each of these categories and are described in detail below.

### Category 1: low level of activity in acute care

#### Boredom

Patients discussed the lack of social and cognitive activities that occurred in the hospital. Patients often described being bored, noting a lack of stimulating activities, especially for those with limited mobility. In order to keep their minds active while in hospital, patients felt that they had to be self-dependent. They described rotating through activities such as knitting, reading, playing on electronic devices such as iPads, watching television, and looking out their room windows; however, none of these activities sufficiently occupied their time. Patients also explained how activities to pass the time, such as watching television, required out-of-pocket payment, which was difficult for those with limited income. Difficulties remaining socially and cognitively active were especially prevalent for patients who were experiencing a prolonged hospital stay. Caregivers shared patients’ concerns about keeping one’s mind active while in hospital.

My concern at this point, is not so much about her medical care, it’s more about making sure that she keeps her spirits up, right, has something to do… [George, Caregiver, Delay in Discharge]

#### Depression

In addition to boredom, patients described feeling depressed by their current hospitalization and lack of activities. For example, a patient with a hip fracture described how he wanted his mental well-being to be checked on while in hospital:

“I think that the single biggest thing that I needed when I was in the hospital… was my emotional distress was kept to myself and if I had had somebody that I could talk to or who was checking on my mental wellbeing… and I’m not talking a psychiatrist or something like that. I’m talking a soft shoulder to talk to type of thing… the hospital looked after me physically and did a good job at it. I can’t complain. But the mental side of it…” [Ronald, Patient, Hip Fracture]

Another patient with hip fracture said:

“it’s depressing… there’s times I looked at that corner, I could just go over there and crawl up in a ball and just die...” [Sarah, Patient, Hip Fracture]

Caregivers and providers also acknowledged the impact of hospitalization on patients’ mental health. Caregivers described their concerns with patients not being themselves and showing limited interest in activities they used to enjoy, and providers explained how the lack of recreational therapy can contribute to depression.

“We have no rec [recreational] therapy or often they get very bored and become depressed even more…” [Jessica, Provider]

#### Lack of physical activity

A lack of physical activity was discussed in terms of the type and availability of therapy and in the willingness of patients to engage. Occupational and physical therapists explained that it was difficult to provide comprehensive rehabilitation due to the acute nature of a hospital. Decision-makers (clinical managers, regional leads) shared the belief that patients were not sufficiently activated in acute care: “we don’t move them in hospital, we don’t mobilize…” [Hannah, Decision-Maker]. A clinical manager felt that patients with a delayed discharge received fewer activities because rehabilitation staff prioritized activating the more “acute person” [Mitchel, Decision-Maker]. Providers and decision-makers talked about their inability to quickly transition patients from acute care into a rehabilitation setting, further contributing to patients’ physical decline. Discharge planners described advocating to facilitate quick transfers out of acute care. However, when quick transfers out of acute care could not occur, patients often deconditioned, which further impacted their care transitions (e.g., could not return home because of stairs).

Similarly, many caregivers felt that there was insufficient physical activity in the acute care setting. Caregivers were concerned about patients’ inabilities to transfer from their bed, stand, walk, toilet, and feed themselves while in hospital. Caregivers described patients needing to learn to walk again “… she hasn’t been on her feet in over four weeks...” [Karen, Caregiver, Delay in Discharge] and expressed disappointment that individuals were not mobilized earlier. Caregivers feared patients losing their ability to walk because this would impact the patients’ ability to return to their pre-hospitalization residence. Patients had mixed views on the appropriateness of the level of physical activity in acute care. While some patients described being satisfied with their physical therapy, others felt disappointed by what they perceived to be limited physical activity.

#### Lack of motivation

Many patients described motivational challenges related to rehabilitation, with inner tensions of knowing the need to participate in physical therapy and feeling unable to participate due to factors such as pain, lack of energy, fatigue, sleep disturbances, and other health complications. Patients explained that despite providers’ attempts to motivate them, they were sometimes still unable to partake in physical therapy. As a patient experiencing a delayed discharge described:

“They really tried to, they tried to motivate you as much as they could but physically, I didn’t have the energy. I didn’t have the energy to do any of the exercises that they wanted me to do. And I couldn’t do it...They kind of expected more out of me and I’m sorry, I was a complete failure to them.” [Dawn, Patient, Delay in Discharge]

Physical therapists talked about the importance of motivating patients, and used positive and negative reinforcement in attempt to motivate patients. These techniques were frequently described as being effective for engaging patients, but physical therapists discussed the importance of tailoring the type of motivation based on what works for each patient.

### Category 2: tensions around patient identity changes and care transitions’ uncertainty

#### Patient identity - things are different

Many patients struggled with a disruption to their personal identity caused by deconditioning. Patients reflected on the activities that they enjoyed before hospitalization and worried about the possibility of no longer being able to participate. A patient with a hip fracture and delayed discharge explained the difficulties coming to terms with this idea:

“But it’s hard to, it’s very, very hard to face up to the facts and I can understand how people gradually go down, I think, being very hard to face up to the fact that they can’t do what they did before.” [Brianne, Patient, Hip Fracture and Delay in Discharge]

While in hospital, patients described the inability to be active and participate in their usual hobbies, which often altered their view of themselves. Patients also reflected on the psychological impact of deconditioning. For example, when reflecting on their current circumstances in hospital, patients used words such as ‘demoralizing’, ‘frustrating’, ‘deteriorating’, and ‘hard’.

#### Uncertainty around care transitions

Second to patients’ shifting perception of self, the most commonly discussed impact of deconditioning was the uncertainty around where the patient would go next. Caregivers and patients mostly wanted a return to the pre-hospitalization residence; however, due to deconditioning, it was unlikely this transition would occur. In some these cases, there was also uncertainty around whether rehabilitation centres or long-term care homes would accept the patient. The challenge of finding facilities that would accept patients, especially those with cognitive or mental health conditions, was acknowledged by all participant types. Finding a post-hospital destination seemed to be more challenging for patients who did not have caregivers, were lower income, or who spoke English as a second language. This uncertainty was articulated by a participant caring for a patient with a hip fracture and delayed discharge:

“…if [Assisted Living Home] can’t take her back and [Rehabilitation Center] said no, like where does she go? Like will they toss her out onto the street? [Assisted Living Home] clearly can’t take her, let’s say because of her limitations. Does she go into like a holding pattern? Does she stay there until we can find some place as an emergency? Like I don’t know, no one can give me any answers.” [Olivia, Caregiver, Hip Fracture and Delay in Discharge]

Patients and caregivers described being pushed out of the hospital before they were ready. A patient in the urban region described being sent to in-patient rehabilitation only to be readmitted to acute care shortly afterwards because she was not “rehab ready” [Diana, Patient, Hip Fracture]. A patient described her discharge experience :

“… a person came into my room. I didn’t know who she was. Never saw her before… she had my chart and she said, ‘I see you’re doing very well. I think you could go home.’ I said, ‘No, I can’t go home.’ I said, ‘I can’t put my foot on the floor.’ And you know, ‘Well’, she said, ‘You don’t think you can manage?’ I said, ‘No, I can’t manage. I can’t go home. I can’t walk.’ So, anyway when she left, I asked the next nurse who was she. And she said, ‘That’s the charge nurse and today is Friday and this is the day that they’re looking for beds.’” [Cynthia, Patient, Delay in Discharge]

### Category 3: physical and social context

#### Social factors - role of roommates, volunteers and providers

The majority of social interactions in hospital occurred between patients and their caregivers or roommates because patients were almost always in their rooms, instead of in more communal social spaces. Participants discussed the role of roommates, who had both positive and negative impacts, on social activities. Positive experiences with patients’ roommates were described, with some patients referring to their roommates as great company and companions who helped pass the time. Providers explained that while shared rooms could improve social interactions, they could sometimes create problems when “there’s just not very good meshing… or their habits are annoying” [Emily, Provider]. One caregiver described negative experiences with her spouse’s roommate, which resulted in her not wanting to visit and her husband not interacting with his roommate.

Additionally, many participants discussed the ability of volunteers to promote positive social interactions in an acute setting. For example, the Hospital Elder Life Program (HELP), a program designed to prevent decline of older hospitalized patients, was discussed [33]. Multiple providers described the importance of HELP in promoting social interaction among hospitalized patients; however, patients and caregivers did not discuss interacting with volunteers. Instead, caregivers expressed disappointment with the lack of volunteer support in acute care.

“But they [volunteers] could certainly go around and just ask people like how is their day doing, there are many patients who don’t have family nearby that are quite lonely, right?... how can we better harness all the social capital, you know, the human talent all around us?” [George, Caregiver, Delay in Discharge]

Social activities were also discussed by participants around interactions with providers. While patients had varying experiences, they generally did not perceive social interactions to be within the job description of providers. All stakeholder types discussed insufficient staffing in acute care. Patients and caregivers seemed resigned and sympathetic towards this limitation, despite being helplessly reliant on staff for activities ranging from toileting to retrieving desired objects (e.g., iPads, books, television remote). Similarly, providers and decision-makers sometimes felt unable to meet the needs of their patients due to system constraints. For example, a provider explained that due to limited staffing, it was challenging to get patients out of bed to eat their meals.

“You know, even a task such as a simple as trying to get them [patients] up for every meal, which you shouldn’t have to have your meal in bed and geriatrics say, get up for meals. It’s easy enough to say, but it’s then going on a workload issue and they have to be monitored while they’re up.” [Jessica, Provider]

#### Physical factors – sub-optimal environment

Participants described a loud hospital environment that was non-conducive to patients’ recovery and overall wellbeing. Lack of sleep was commonly discussed by patients, caregivers, and providers in both regions. Impact on sleep was especially prevalent in the rural hospital, where shared hospital rooms were open concept with thin curtains drawn around each bed. Participants described being prescribed sleeping medications to alleviate sleeping issues. The most common complaints across both regions were about patients wandering the halls, noises from other patients on the hospital unit (roommates or otherwise), and noises from hospital machines, all of which impacted patients’ ability to get adequate sleep.

“…one woman came in because she had pneumonia and they had to move her to a separate room and lock the door so that she couldn’t get out because she kept wandering out. And of course, she still had pneumonia so nobody wanted to be anywhere near her. It didn’t seem appropriate at all and there were quite a few [patients] who were howling in the night and this sort of thing and then bells kept going off in the night and my sleep was definitely very badly affected.” [Jen, Patient, Hip Fracture]

Patients, caregivers, and providers discussed the negative impact that poor sleep had on physical, cognitive, and social activities. Specifically, caregivers described patients missing their physical rehabilitation because they were sleeping during the day, which was often attributed to poor sleep at night or to medication side effects.

“… most of the time he’s sound asleep. And even when she [physical therapist] comes in to do exercise, if he’s sleeping, you can’t do anything.” [Flora, Caregiver, Hip Fracture and Delay in Discharge]

## Discussion

In our qualitative study, we explored the experiences of multiple stakeholders with physical, cognitive, and social activities in hospital. We included experiences of a rural and an urban region in Ontario, Canada. Despite anticipating varying experiences between participants in the rural and urban regions based on differing geographic setting and allocations of resources, no substantive differences in experiences were found, thus results were presented together. Our findings highlighted a lack of physical, social, and cognitive activities in acute hospital, which contributed to HAD. Deconditioning impacted patients and caregivers by increasing stress and anxiety and imposed overall challenges with transitions in care.

Capturing perspectives from patients, caregivers, providers, and decision makers provided a multi-dimensional perspective of HAD. Patients and caregivers were yearning for physical, social, and recreational activities to address their mental health and functional needs, while providers struggled to provide holistic care in acute environments. Our research raises an important question of *how do we address HAD within models of care and organizational contexts that are designed for acute and medical-oriented care?* The care provided by caregivers was paramount in addressing these critical care gaps, but questions remain as to whether or not this is sustainable or equitable; particularly since not all patients will have access to a caregiver who can provide these supports. Providers acknowledged the importance of socially-oriented programs (including care provided by volunteers) but that these programs are often undervalued as evidenced by being vulnerable to hospital budget cuts.

Biopsychosocial dimensions of care need to be addressed simultaneously to prevent HAD. More specifically, in our study we identified that patients and caregivers were fearful of functional decline, with consequential impacts to self-identity and mental health. Providers also witnessed patients who were reluctant and fearful to engage in physical rehabilitation, showing the interconnectedness of physical and mental health. Patients and caregivers placed substantial emphasis on the ability to walk and expressed concerns that if walking targets were not met, they would not be able to return home (which was often the preferred location). Further, our findings highlighted frustrations with the lack of promotion of independence as well as the lack of availability and intensity of rehabilitation. Recently, Bender and Holyoke identified similar challenges with promotion of independence in their case study of six Canadian hospitals [11]. The case study involved interviews with experts in home and community care who were involved in reviewing practices around delays in discharge of the six hospitals. The researchers identified that deconditioning occurred mostly as a result of ‘institutionalized care’ and hospital staffs’ overall aversion to risk. Moreover, they concluded that the underestimation of patients’ abilities and minimal encouragement of patients’ taking on some activities of daily living while in acute care (e.g., dressing, feeding) further contributed to deconditioning.

Our findings also identified a lack of cognitive and social activities. Specifically, caregivers described experiencing substantial burden by frequently visiting due to hospital staff being too busy or focusing on patients with more urgent needs. While some caregivers and patients seemed to accept the health system constraints and limited staffing resources, participants spoke about the need to leverage more appropriate resources such as volunteers and recreational therapists. Our findings reinforce the impact of both physical and psychological harm, and the importance of meaningfully engaging with patients and caregivers to advance patient safety and minimize HAD [34]. Meaningful engagement with patients and caregivers must include what matters most to them during the hospital stay and care transitions. These principles align the much broader definition of safety articulated by Vincent and colleagues through the Measuring and Monitoring Safety Framework, which denotes that safety is much more than the minimization of physical harm. Safety needs to consider psychological and emotional health of patients and families, have a focus on prevention, and include processes that are in place to create safer and more reliable care experiences [35, 36].

Based on our study findings and previous literature [12, 15, 16, 37], we provide several recommendations to address HAD and improve patient safety, which also map onto core components of transitional care outlined by Naylor and colleagues [38]: (1) measuring physical and psychological function and well-being throughout hospitalization in order to identify if deconditioning is occurring and address it early [15]; (2) redesigning hospital environments (e.g., private patient rooms and social spaces) to encourage social activities and promote safe social interactions; (3) leveraging existing resources in a more meaningful way (volunteer sector and family support) to ensure physical, social, and cognitive activities are routine while in hospital; (4) increasing access to rehabilitation and recreational therapy during the patients’ acute hospital stay to limit physical deconditioning while waiting for the next point-of-care; (5) encouraging tailored social and cognitive stimulation in hospital (according to the patient’s circumstances and preferences) to limit cognitive decline; and (6) measuring patient reported outcomes and patient/caregiver experiences. Currently in Canada, functional status is not routinely or comprehensively measured in acute care; therefore, there are limited quantitative data on HAD to inform health services delivery.

There are a few limitations to this study. Firstly, despite significant efforts, we had limited participation from hospital physicians. Secondly, we had limited the diversity of our participants (e.g., most English speaking), which may limit the transferability of our results. Future research would be warranted to gain more physician perspectives on how to improve HAD, as well as a more diverse group of participants to explore how social location (e.g., language, ethnicity, income) may influence experiences with HAD.

## Conclusions

Despite efforts to improve patient safety, hospital-based patient harm and deconditioning remains problematic [4–6]. In this study, we explored participants’ perspectives and experiences with HAD. We identified substantial experiences with physical, cognitive, and social inactivation, which, from the perspectives of patients, caregivers, providers, and decision-makers led to deconditioning. These experiences with HAD often contributed to delays in discharge and resulted in additional care resources offered at the next point of care. By capturing the perspectives of patients, caregivers, providers, and decision makers, our findings highlighted different sets of tensions that need attention if HAD is to be appropriately addressed (e.g., patients and caregivers needing ongoing therapy that addresses their physical, social, and mental health needs and providers having the flexibility in acute environments to attend to these needs). Specifically, our findings support meaningful patient and caregiver engagement to minimize HAD, which includes several of the following: measuring physical/psychological function and well-being throughout hospitalization; redesigning hospital environments; and increasing access to rehabilitation during acute hospital stay while patients wait for the next point-of-care.

## Data Availability

The datasets generated and/or analysed during the current study are not publicly available in order to protect the identity of participants, but de-identified information is available from the corresponding author on reasonable request.

## Abbreviations

HAD: Hospital Associated Deconditioning
HELP: Hospital Elder Life Program
